# Measuring preferences for meaningful activity in assisted living residents with dementia

**DOI:** 10.1002/alz.086605

**Published:** 2025-01-09

**Authors:** Sorah Levy

**Affiliations:** ^1^ University of Maryland Baltimore, Baltimore, MD USA

## Abstract

Residents with dementia living in assisted living settings report that participating in activities contributes to their psychosocial well‐being and evidence suggests it has the potential to address outcomes such as improved mood, reduced agitation, and increased feelings of social connection. However, assisted living residents with dementia are infrequently asked about their preferences and interests for activities and those preferences might change over time along with their cognitive or physical abilities. Further it can be challenging for staff to focus on engaging residents in meaningful activity when they have competing demands to perform other care responsibilities. There is a need for practical tools to help assisted living staff evaluate preferences and identify opportunities for meaningful activity for residents with dementia that are tailored to those preferences. The purpose of this study was to develop a measurement tool to evaluate preferences and establish goals for meaningful activity, the Assessment of Resident Preferences for Meaningful Activity Tool. This tool was developed specifically for assisted living residents with dementia and collects brief information about the resident’s background (e.g. where they grew up, what they did for work, hobbies etc.) and includes a list of short answer prompts about what is meaningful to the resident (e.g. “I feel good about myself when…”, “I feel accomplished when…”, “I feel a sense of joy when…”). The information collected in this tool is used to develop an individualized plan for engaging the resident in meaningful activity. The tool was completed with assisted living residents with moderate to severe dementia enrolled in the randomized controlled trial, Meaningful Activities for Managing Behavioral Symptoms of Distress (MAC‐4‐BSD). A description of the tool will be presented along with key strategies to facilitate the implementation of this tool in practice for engaging residents with dementia in meaningful activity.

